# Notch1 directly induced CD133 expression in human diffuse type gastric cancers

**DOI:** 10.18632/oncotarget.10967

**Published:** 2016-07-30

**Authors:** Hidetomo Konishi, Naoki Asano, Akira Imatani, Osamu Kimura, Yutaka Kondo, Xiaoyi Jin, Takeshi Kanno, Waku Hatta, Nobuyuki Ara, Kiyotaka Asanuma, Tomoyuki Koike, Tooru Shimosegawa

**Affiliations:** ^1^ Division of Gastroenterology, Tohoku University Graduate School of Medicine, Aoba-ku, Sendai, Miyagi 980-8574, Japan

**Keywords:** Notch, RBP-J kappa, cancer stem cell, gastric cancer, CD133

## Abstract

CD133 is considered as a stem-like cell marker in some cancers including gastric cancers, and Notch1 signaling is known to play an important role in the maintenance and differentiation of stem-like cells. We aimed to investigate whether Notch1 signaling contributes to the carcinogenesis of gastric cancers and CD133 induction. CD133 expression was detected in 51.4% of diffuse type gastric cancers while it was not detected in intestinal type gastric cancers. Similarly, only poorly-differentiated gastric cancer cell lines expressed CD133 and activated-Notch1. Inhibiting Notch1 signaling resulted in decreased CD133 expression, side population cells, cell proliferation and anchorage independent cell growth. Chromatin immunoprecipitation suggested that this Notch1 dependent regulation of CD133 was caused by direct binding of activated-Notch1 to the RBP-Jκ binding site in the 5′ promoter region of *CD133* gene. In addition, knocking down RBP-Jκ reduced CD133 induction in activated-Notch1 transfected cells. These findings suggested that Notch1 signaling plays an important role in the maintenance of the cancer stem-like phenotype in diffuse type gastric cancer through an RBP-Jκ dependent pathway and that inhibiting Notch1 signaling could be an effective therapy against CD133 positive diffuse type gastric cancers.

## INTRODUCTION

Gastric cancer is the fourth most common cancer in the world [1], and can be classified into two histological groups: intestinal type and diffuse type [2–4]. Intestinal type gastric cancers arise from gastric mucosal atrophy and intestinal metaplasia, which are both caused by chronic *Helicobacter pylori* infection [5]. In contrast, diffuse type gastric cancers are considered to arise from an abnormality of stem-like cells in a proliferating zone in the isthmus of oxyntic glands [3]. These diffuse type gastric cancers progress more rapidly and easily cause metastasis leading to poor prognosis [6].

Notch signaling has been reported to be involved in the maintenance and differentiation of stem-like cells [7, 8]. It is also involved in the development of adenomas and cancers [9, 10].

On the other hand, CD133 (or Prominin1) is considered as a stem-like cell marker in various cancers including gastric cancers [11–15].

In this study, we aimed to investigate the expression of the stem-like cell marker CD133 in gastric cancers, and to clarify the role of Notch1 pathway on cell proliferation, tumorigenesis and induction of CD133 in diffuse type gastric cancers.

## RESULTS

### CD133 was expressed in diffuse type gastric cancers

**Figure 1:**
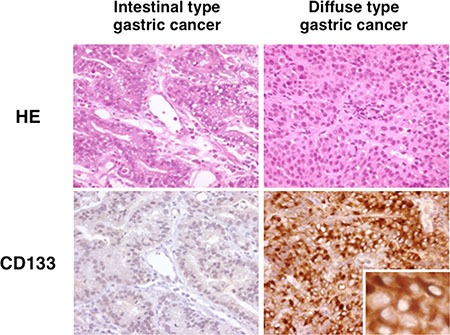
CD133 expression in human gastric cancers Representative histological sections (HE) of intestinal type and diffuse type gastric cancer. Staining for CD133 was not observed in intestinal-type gastric cancer cells, whereas it was observed in a membranous distribution in diffuse-type gastric cancer cells. *Insert* shows higher magnification where CD133 is localized in the diffuse-type gastric cancer cells.

First of all, we investigated CD133 expression in gastric cancer specimens by immunohistochemistry. As shown in Figure 1, CD133 was expressed mainly on the membrane of diffuse type gastric cancer cells but not on that of intestinal type gastric cancer cells. The specificity of the antibody was confirmed by staining CD133 positive KATOIII cells and negative AGS cells (data not shown). Among the 74 specimens we evaluated, CD133 was expressed in 19/37 diffuse type gastric cancers (51.4%), whereas it was not expressed in any of the intestinal type gastric cancers (0/37; 0%). The average proportions of positive cells were 69.7% and 1.7% in CD133 positive and negative diffuse type gastric cancers, respectively. There was no significant difference between the CD133 positive and negative diffuse type gastric cancers in terms of gender, age and histology.

These data suggested that CD133 is expressed in diffuse type gastric cancers but not in intestinal type gastric cancers.

### Activated-Notch1 and CD133 were expressed only in poorly-differentiated gastric cancer cell lines

**Figure 2:**
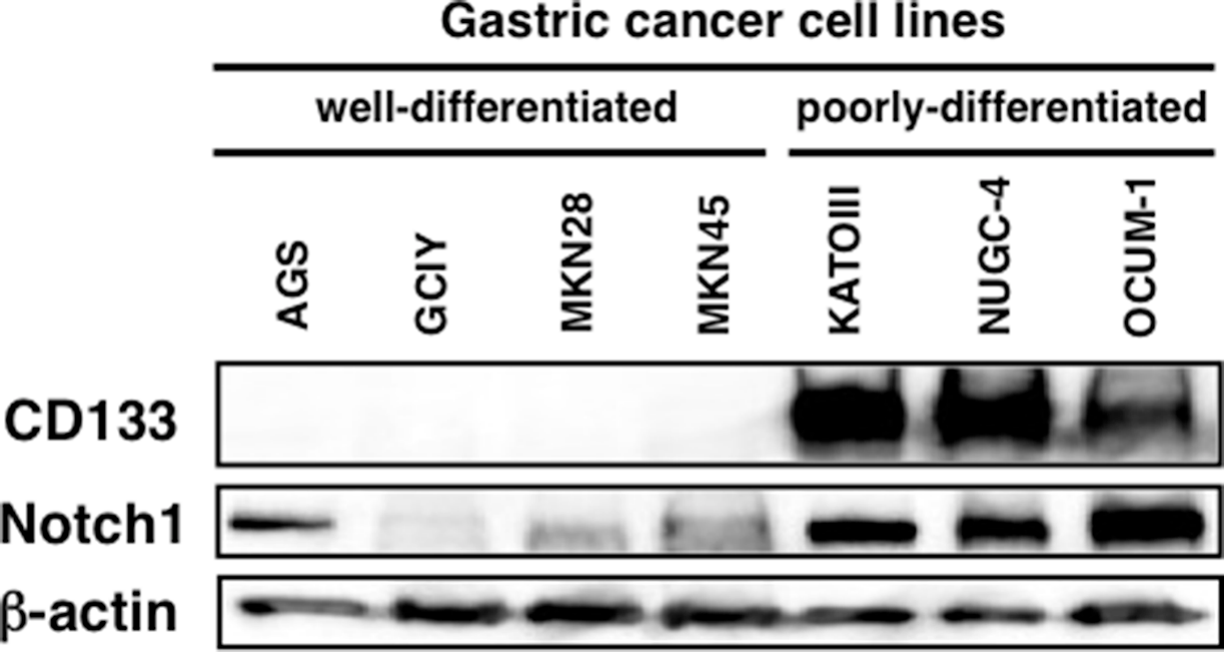
Cleaved Notch1 and CD133 protein expression in human gastric cancer cell lines Poorly-differentiated gastric cancer cell lines expressed both cleaved Notch1 and CD133.

We then investigated the expression of CD133 and activated-Notch1 (N1ICD) in gastric cancer cell lines to find out whether the finding mentioned above could be seen in these cell lines. As shown in Figure 2, both N1ICD and CD133 were expressed only in non-adherent poorly-differentiated gastric cancer cell lines (KATOIII, NUGC-4, OCUM-1).

### Knocking-down Notch1 signaling reduced CD133 expression

**Figure 3:**
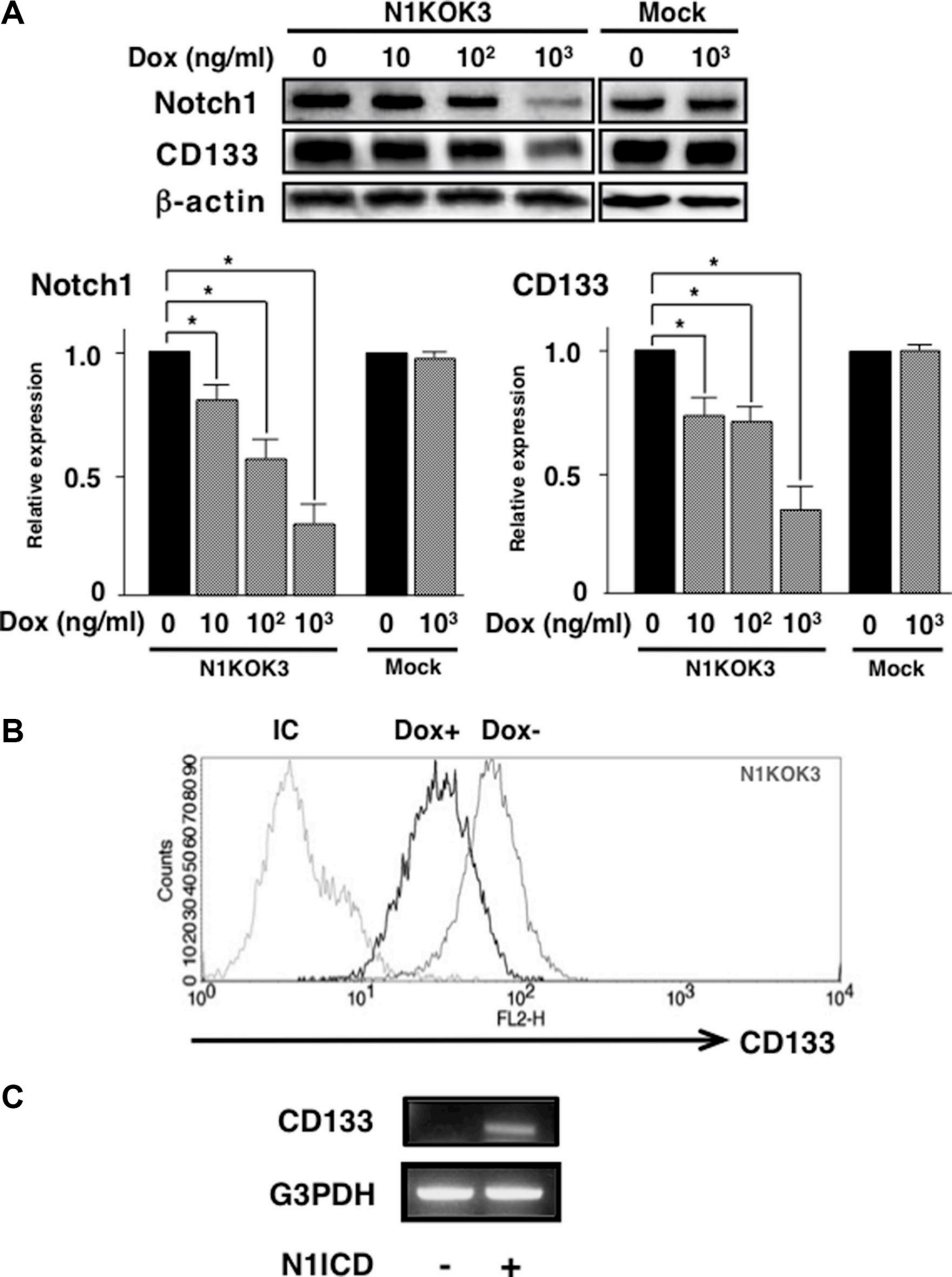
Silencing Notch1 led to reduced CD133 expression NIKOK3 cells, which induced Notch1 shRNA by Dox were generated from KATOIII cells. (**A**) N1KOK3 cells and Mock cells were stimulated with the indicated doses of Dox. Cleaved Notch1 and CD133 expression was evaluated by western blot. Protein expression was quantified by densitometry. All experiments were performed at least thrice, and the results are shown as means ± S.E. *P < 0.05 compared to Dox unstimulated cells. (**B**) CD133 expression in N1KOK3 cells stimulated with or without Dox was assessed by flow cytometry. (**C**) CD133 expression in N1ICD transfected AGS cells. AGS was transfected with N1ICD-expressing vector, and CD133 expression was assessed by RT-PCR.

Since both N1ICD and CD133 were co-expressed in poorly-differentiated gastric cancer cell lines, we hypothesized that CD133 expression is induced by Notch1 signaling. To assess this hypothesis, we developed a gastric cancer cell line whose Notch1 could be knocked-down by addition of doxycycline (Dox) to the culture (N1KOK3; see Methods). As shown in Figure 3A, increasing concentrations of Dox led to reduced N1ICD and CD133 expression in N1KOK3 cells, whereas it had no effect on Mock cells. 10^3^ ng/ml Dox decreased Notch1 expression to 0.24 ± 0.13 folds and CD133 expression to 0.30 ± 0.19 folds. This result was also confirmed by flowcytometric study, where CD133 expression on the cell surface was decreased in response to Notch1 inhibition by the addition of Dox (Figure 3B).

Then we investigated whether this CD133 induction by Notch1 occurred in other cells as well. Accordingly, we transfected AGS cells (which don’t express CD133) with N1ICD and assessed CD133 expression. As shown in Figure 3C, transfection of N1ICD induced CD133 expression in AGS cells. This result suggested that CD133 expression is induced by Notch1 signaling.

### CD133 expression was up-regulated by Notch1 signaling through RBP-Jκ

**Figure 4:**
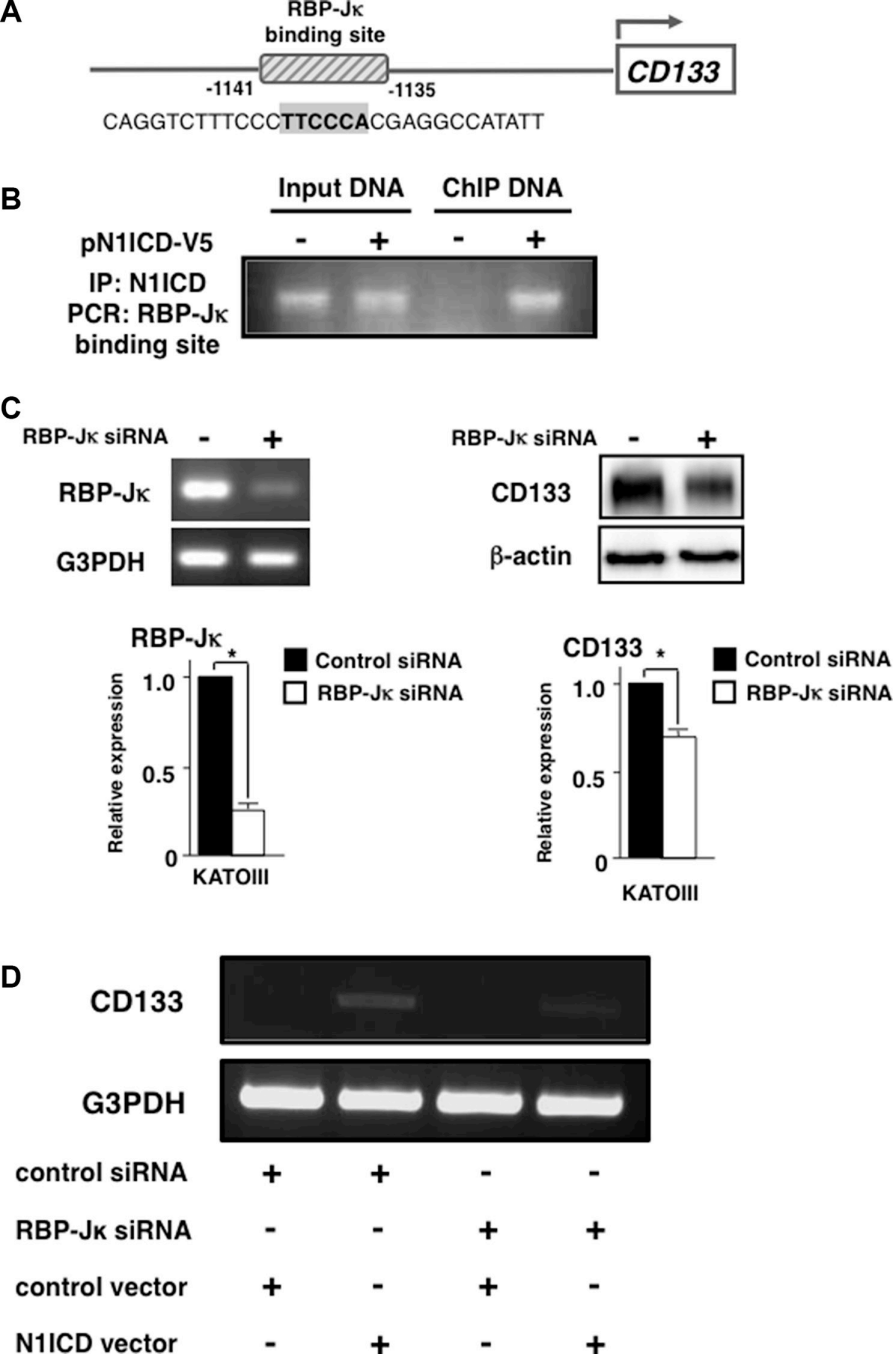
Activated-Notch1 binds to RBP-Jκ binding site in *CD133* promoter (**A**) Computer analysis revealed a RBP-Jκ binding site in *CD133* promoter region. The putative binding site is indicated in bold. (**B**) KATOIII cells were transfected with either empty vector or pN1ICD-V5. Cross-linked chromatin was immunoprecipitated with anti-V5 antibody and analyzed by PCR using primers that flank the RBP-Jκ binding site. (**C**) KATOIII cells were transfected with RBP-Jκ siRNA. (*left panel*) RBP-Jκ expression was assessed by RT-PCR. (*right panel*) CD133 expression was evaluated by western blot. Densitometric analysis of RBP-Jκ and CD133 was performed. (**D**) AGS cells were transfected with either control siRNA or RBP-Jκ siRNA followed by either the control empty vector or N1ICD expressing plasmid transfection. CD133 and G3PDH expression was evaluated by RT-PCR. All of these experiments were performed thrice. The results are shown as means ± S.E. *P < 0.05 compared to control cells.

Above results suggested that Notch1 signaling induced CD133 expression. We then investigated the mechanism involved in this regulation. By using MatInspector software, we found an RBP-Jκ binding motif in the –1141 to –1135 region of the 5′ promoter region of *CD133* gene (Figure 4A). Since RBP-Jκ is a known motif that binds N1ICD, we speculated that CD133 expression was regulated by N1ICD binding to this site. As shown in Figure 4B, chromatin immunoprecipitation revealed that N1ICD does bind to this RBP-Jκ binding motif in the *CD133* promoter. To verify this result, we investigated if knocking down RBP-Jκ altered CD133 expression. As shown in Figure 4C, RBP-Jκ specific siRNA significantly reduced CD133 expression to 0.72 ± 0.04 folds. These results suggested that N1ICD directly induced CD133 expression through RBP-Jκ. To further substantiate these results, we investigated whether silencing RBP-Jκ blocked N1ICD-induced CD133 up-regulation in AGS cells. As shown in Figure 4D, CD133 was induced in AGS cells transfected with N1ICD expressing vector, while this expression was reduced by RBP-Jκ siRNA co-transfection. Taken together, these results strongly suggest that CD133 induction by N1ICD is dependent on RBP-Jκ.

### Down-regulation of Notch1 reduced side population cells

**Figure 5:**
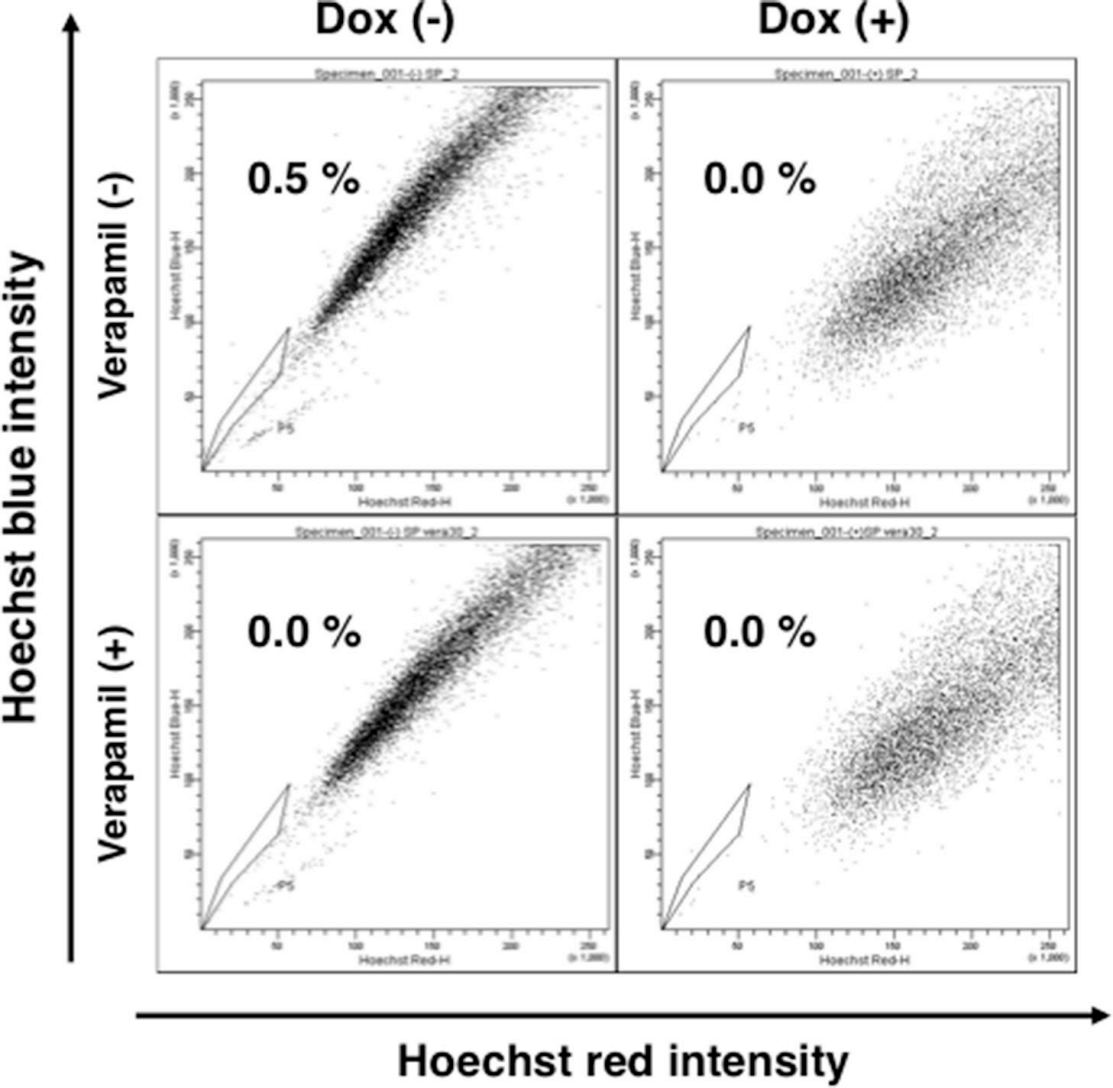
Silencing Notch1 abolished side population cells NIKOK3 cells were cultured with or without Dox for 72 hours, and then were stained with Hoechst 33342 in the presence or absence of verapamil.

CD133 is regarded as one of the stem-like cell markers. On the other hand, side population (SP) cells are identified as cell population that possess ATP-binding cassette (ABC) transporters, which can pump out the passively uptaken Hoechst 33342 dye. These cells express stem-like cell markers and possess the ability to generate non-SP cells [16, 17], and they are considered as multi-potential stem-like cells for these characteristics. Based on the finding that silencing Notch1 reduced stem-like cell marker CD133, we decided to look at the effect of Notch1 on SP cells. We performed a flowcytometric analysis for SP cells with N1KOK3 cells in the presence and the absence of Dox and verapamil. As shown in Figure 5, N1KOK3 cells without Dox contained 0.5% of Hoechst 33342-excreting SP cells. Addition of verapamil, the ABC transporter inhibitor, confirmed this population as SP cells. These SP cells were abolished in the presence of Dox, that is, by the down-regulation of N1ICD. Together with the result of CD133, this result suggested that N1ICD induced the stem-like cell phenotype.

### Down-regulation of Notch1 reduced anchorage independent cell growth

**Figure 6:**
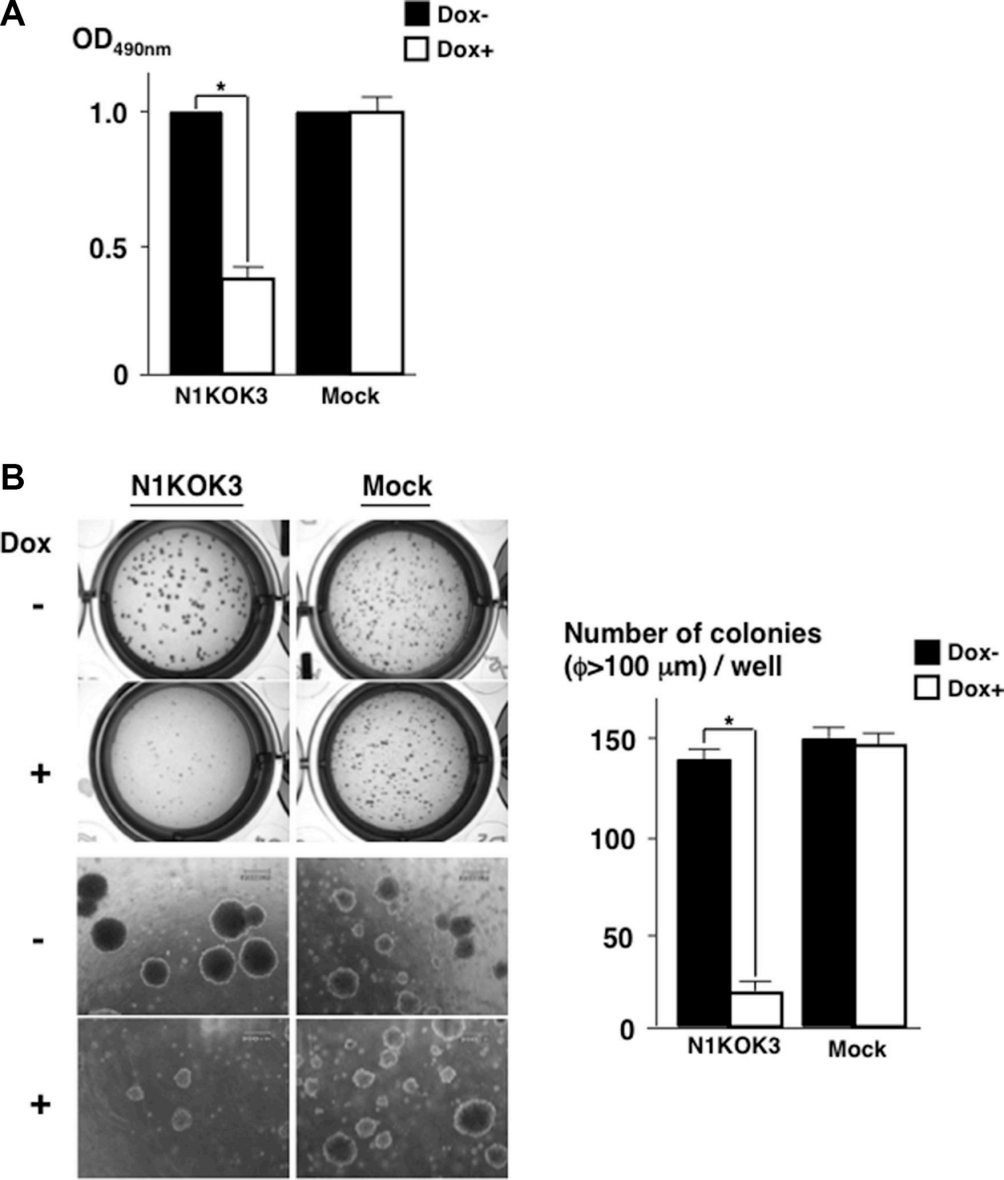
Silencing Notch1 reduced cell proliferation and anchorage independent cell growth (**A**) NIKOK3 cells were cultured with or without Dox for 72 hours, and cell proliferation was evaluated by MTT assay. (**B**) Soft agar assay was performed with N1KOK3 cells cultured with or without Dox for 4 weeks. The colonies over 100 μm in diameter were counted. All experiments were performed thrice, and the results are shown as means ± S.E. *P < 0.05 compared to Dox un-stimulated cells.

Since down-regulating Notch1 reduced SP cells and CD133, we then investigated the proliferation of Notch1-silenced cells. As shown in Figure 6A, down-regulation of Notch1 reduced the proliferation to 0.43 ± 0.03 folds. We also found out that silencing RBP-Jκ also reduced cell proliferation (data not shown). Next, we evaluated the anchorage independent cell growth by soft agar assay. As shown in Figure 6B, the number of colonies in Notch1 silenced N1KOK3 cells (8.33 ± 2.42) was reduced significantly compared to that of Notch1 expressing N1KOK3 cells (142.5 ± 4.72) (*P* < 0.01). On the other hand, the addition of Dox to the culture did not affect the number of colonies in Mock cells (148.8 ± 5.00 and 148 ± 5.40, *P* = 0.786). This result clearly showed that silencing Notch1 reduced the number of colonies, suggesting reduced anchorage independent cell growth.

## DISCUSSION

In this study, we discovered that stem-like cell marker CD133 was expressed in human diffuse type gastric cancers and that Notch1 directly regulated its expression in an RBP-Jκ dependent manner. Furthermore, our results suggested that Notch1 signaling is required for maintaining the stem-like cell population, proliferation and anchorage independent growth in gastric cancers.

Immunohistochemistry showed that CD133 was expressed in half of the diffuse type gastric cancers, while it was not expressed in any of the intestinal type gastric cancers.

In addition to CD133, Notch1 is also considered to be a molecule essential for the maintenance and differentiation of stem-like cells [7]. Indeed our result showed that poorly-differentiated gastric cancer cells expressed not only CD133 but also activated-Notch1. These findings suggested that a part of diffuse type gastric cancers arise directly from CD133 positive stem-like cells through the activation of aberrant Notch signaling.

As for the mechanism involved in the regulation of CD133, we showed in this study that activated-Notch1 binds to the RBP-Jκ binding site in the 5′ promoter of CD133 by chromatin immunoprecipitation, and that silencing RBP-Jκ reduced CD133 expression. These results strongly suggest that CD133 induction by Notch1 is dependent on RBP-Jκ.

Previous studies have also reported on the correlation between Notch1 and CD133 in other types of cancers such as glioma, breast cancer, lung cancer and prostate cancer [18–21]. However, the present study stands out from these previous reports in that we showed that activated-Notch1 directly induced CD133 through an RBP-Jκ dependent pathway. On the other hand, knocking down Notch1 or RBP-Jκ did not completely abrogate CD133 expression, and it is possible that other signaling pathway is involved in the induction of CD133. Recent report showed that IL-6 activated Notch3 signaling also induced CD133 overexpressing cells in breast cancer and further investigations are warranted [22].

Silencing Notch1 not only reduced CD133 expression, but also abolished SP cells, a subset of cells that are considered to have a stem-like cell phenotype. Therefore, this is another evidence showing that Notch1 was required for the maintenance of stem-like cells. These stem-like cells are considered to initiate cancers in various organs [23]. Indeed in our study, silencing Notch1 signaling, which reduced the stem-like cell phenotype, led to decreased cell proliferation and reduced anchorage independent growth. This suggested that Notch1 signaling plays an important role in the carcinogenesis and the maintenance of CD133 positive diffuse type gastric cancers.

In our study, the expression of CD133 was limited to diffuse type gastric cancers. This was in accordance with previous study where poorly-differentiated gastric adenocarcinomas expressed more CD133 than well-differentiated gastric adenocarcinomas, and CD133 expression increased with the increase in the malignancy grades of gastric cancer [24]. On the contrary, there was also previous report indicating that CD133 was expressed in well-differentiated gastric cancers and not poorly-differentiated gastric cancers, although CD133-positive gastric cancers had significantly worse prognosis [25]. Further studies are warranted to elucidate the cause of this discrepancy.

Recently reported meta-analysis showed that overexpression of CD133 in gastric cancer was associated with lymph node and distant metastasis, poor TNM stage and poor prognosis [26]. Therefore, it is possible that CD133 positive cells play an important role in carcinogenesis and maintenance of diffuse type gastric cancers, although it is unclear whether this role depends on CD133. Further studies are warranted to address the function of CD133.

In conclusion, our study showed that Notch1 induced CD133 expression through RBP-Jκ dependent pathway, and Notch1 played a critical role in the tumor progression of diffuse type gastric cancers. Inhibiting Notch1 signaling might be an effective therapeutic target against CD133 positive diffuse type gastric cancers.

## MATERIALS AND METHODS

### Cells

Human gastric cancer cell lines GCIY, NUGC-4 and KATOIII were provided by Cell Resource Center for Biomedical Research, Tohoku University (Sendai, Japan), OCUM-1, MKN28 and MKN45 were purchased from Health Science Research Resources Bank (Osaka, Japan), and AGS was purchased from American Type Culture Collection (Manassas, VA). These cells were maintained following the provider's protocols.

### Tissue samples

Thirty-seven diffuse type and 37 intestinal type gastric cancer tissues were obtained by biopsy from 74 patients with gastric cancer who underwent endoscopy in Tohoku University Hospital during August 2008 to January 2009. A written informed consent was obtained from all patients and this study was conducted with an approval from the Ethics Review Board of Tohoku University after obtaining informed consent from the patients.

### Antibodies and reagents

Anti-human CD133 antibody (W6B3C1, 293C3) and mouse IgG were purchased from Miltenyi Biotec, anti-β-actin antibody (AC-74) was from Sigma Aldrich. Antibodies against cleaved Notch1 (Val1744) and CD133 (C24B9) were purchased from Cell Signaling Technology and anti-V5 antibody was from Bethyl Laboratories. Doxycycline (Dox) was from Clontech and G418 was from Roche Diagnostics.

### Western blot analysis

Cell lysates were prepared with lysis buffer (0.05 M Tris-HCl (pH 7.5), 0.15 M NaCl, 1% Triton X, 0.1 mM DTT, 1 × TBS supplemented with Complete Mini (Roche)). 10 μg of cell lysates were separated on 10% Tris-glycine polyacrylamide gel (Bio-Rad) by electrophoresis and transferred onto polyvinylidene difluoride membrane (Millipore). After blocking, the membrane was incubated with primary antibody at 4°C overnight. The membrane was then washed with TBS-T (1 × TBS containing 0.1% Tween 20) and incubated with the secondary antibody at room temperature for an hour. Proteins on the membrane were detected with ECL Advance Reagent (Amersham Biosciences) and visualized by a lumino-image analyzer LAS1000 Plus (Fuji Film).

### Immunohistochemistry

Immunohistochemistry for gastric cancer tissue was performed as previously described [27]. Briefly, specimens were fixed in 10% buffered formalin and embedded in paraffin. Three μm thick serial sections were cut from the paraffin blocks and dewaxed. After inhibiting the endogenous peroxidase activity, the sections were autoclaved in Dako Target Retrieval Solution (Dako Cytomation). The primary antibody against CD133 was added to the sections at 1:225 dilutions, and the sections were incubated at 4°C overnight. The sections were then treated with biotinylated anti-mouse IgG antibody (Nichirei) and incubated for 30 minutes at room temperature. After adding HRP-linked streptavidin (Nichirei), the antigen-antibody complexes were visualized by DAB (Dako). Existence of 20% or more CD133 positive cells among tumor cells was considered as CD133 positive.

### siRNA transfection

siRNA for human Notch1, RBP-Jκ and control siRNA were purchased from Dharmacon. The siRNAs were transfected into KATOIII cells and AGS cells with Lipofectamine 2000 (Invitrogen) following the manufacturer's protocol. Forty-eight hours after transfection, the cells were harvested and subjected to western blot or RT-PCR. In some experiments the siRNA-transfected cells were co-transfected with control empty pcDNA4.0-V5/His vector or cleaved Notch1 expression vector pN1ICD-V5 (gifts from Dr. Doreen Kacer [28]).

### Construction of doxycycline-inducible Notch1 shRNA vector and establishment of stable clone expressing this vector

The most effective sequence of siRNA for Notch1 (5′-GAACGGGGCUAACAAAGAUUU-3′) was determined by western blotting, and sense and antisense oligonucleotides were synthesized accordingly. Annealed oligonucleotides were subcloned into the pSingle-tTS-shRNA vector (Clontech) between the HindIII and XhoI sites. After confirming the sequence, the vector was transfected into KATOIII cells using Lipofectamine LTX (Invitrogen), and the transfected cells were selected using 200 μg/ml G418. Single clones were selected and were stimulated with 10^3^ ng/ml Dox. Then, the cells were harvested and subjected to western blot for Notch1. The clone in which Notch1 was most effectively suppressed was named N1KOK3 and was employed for further studies. KATOIII cells stably transfected with the empty vector were used as a control (Mock).

### Flowcytometric analysis

10^6^ Dox treated or untreated N1KON3 cells were stained with CD133-PE (Miltenyi) and analyzed by FACS Calibur (BD Bioscience). In the side population (SP) cell analysis, N1KOK3 cells were cultured in the presence of Hoechst 33342 with or without 30 μmol/ml verapamil (Sigma). The SP cell fraction was analyzed using FACS Aria II (BD Bioscience).

### Chromatin immunoprecipitation (ChIP) assay

KATOIII cells were transfected with either the empty pcDNA4.0-V5/His vector or the cleaved Notch1 expression vector pN1ICD-V5 (gifts from Dr. Doreen Kacer MMCRI [28]) as previously described [29]. Forty-eight hours after transfection, the cells were harvested, fixed with 1% formalin and subjected to ChIP assay using ChIP-It Express Kit (Active Motif). Chromatin was immunoprecipitated with anti-V5 antibody and PCR was performed using a primer pair specifically designed for the RBP-Jκ binding site in the CD133 promoter: 5′-TTCCTCTTGTCTCAACTGCAAG-3′, 5′-AATCTCAACTACCCAGGGACAC-3′.

### Total RNA isolation and reverse transcription polymerase chain reaction (RT-PCR)

Total RNA was isolated from cells using TRIzol reagent (Invitrogen) following the manufacturer's protocol. cDNA was synthesized from the isolated RNA using Superscript III reverse transcriptase (Invitrogen) and PCR was performed as previously described [30]. The sequences of the primers were as follows: RBP-Jκ 5′- AGAAAGAATAATTCAATTTCAGGCCACTCC ATGTCC-3′, 5′- TTCTGGTGTGTAGGTAAAGGTAA GGCTGGTGG-3′; G3PDH 5′-CAGGTGGTCTCCTCT GACTTCAAC-3′, 5′-AAGGGTCTACATGGCAACTCT GAGG-3′.

### MTT assay

N1KOK3 cells and Mock cells were cultured in 96 well plates for 72 hours with or without 1000 ng/ml Dox. Cell proliferation was measured using CellTiter 96 Aqueous One Solution Cell Proliferation Assay Kit (Promega). The cell proliferation index was calculated as the ratio of the absorbance in relation to that of untreated cells.

### Soft agar assay

In order to assess the anchorage independent growth of the cells, soft agar assay was employed as previously described [31]. Briefly, equal numbers (10^4^ cells/well in 12 well plates) of N1KOK3 cells and Mock cells were placed in soft agar containing 0.34% bacto agar (Difco) and plated onto hard agar containing 0.9% bacto agar. The plate was cultured for 4 weeks with or without 1000 ng/ml Dox. Then the colonies over 100 μm in diameter were counted.

### Statistical analysis

The data were analyzed by student's *t-test* with the use of StatView (SAS Institute), and expressed as mean ± S.E. Statistical significance was assessed by Student's *t-test*. A value of *P* < 0.05 was considered to be statistically significant.
